# Supplementary material for: DeSUMOylation of a Verticillium dahliae enolase facilitates virulence by derepressing the expression of the effector VdSCP8
Source: Nat Commun. 2023 Aug 10;14:4844. doi: 10.1038/s41467-023-40384-w (PMC10415295; doi:10.1038/s41467-023-40384-w)
Supplement: Supplementary file 1 — Supplementary Information [file 41467_2023_40384_MOESM1_ESM.pdf]

This file includes:

Supplemental Methods

Supplemental Figures 1-13

Supplemental Tables 1-3

Supplemental Methods:

Plasmid construction and transformation

To obtain the knockout plasmids pGKO-VdUlpB, pGKO-VdUbc9 and pGKO-VdUlpA, the upstream and downstream genomic sequences of *VdUlpB*, *VdUbc9* (VDAG_00270) and *VdUlpA* (VDAG_10023) were cloned. The corresponding fragments were ligated into the *Pac* I-linearized pGKO binary vector by the In-Fusion cloning method (Vazyme, C113-02) based on homologous recombination, and transformation was performed as described previously to produce the knockout mutants VdΔ*ulpb VdΔubc9 and VdΔulpa* in V592, and VdΔ*ulpb*^{JR2} in VdJR2 strain. Southern blotting was conducted to confirm the deletion of *VdUlpB VdUbc9* and VdΔ*ulpb*^{JR2}. The probes were amplified and labeled with biotin. The primers used above are listed in Supplemental data 1.

The *VdUlpB* gene, including the native promoter and terminator together with the HA fragment, were amplified to generate the complementary plasmid pNEO-VdUlpB-HA, and fragments were ligated into a *Hind* III/*EcoR* I-linearized pNEO binary vector. To obtain the complementary plasmid pNEO-VdUlpBm-HA, the cysteine (C711) in pNEO-VdUlpB-HA was mutated into serine using the Fast Site-Directed Mutagenesis kit (TIANGEN, KM101). To generate the pNEO-VdUlpB-GFP or pNEO-VdUlpBΔN-GFP construct, full-length *VdUlpB* or *VdUlpBΔN* sequences together with the GFP fragment were amplified, and ligated into a *Pac* I/*BamH* I-linearized pNEO binary vector, respectively. The primers used above are listed in Supplemental data 1. pNEO-VdUlpB-HA and pNEO-VdUlpBm-HA constructs were transformed into the WT V592 and knockout mutant VdΔ*ulpb* or Vd$^{T\text{-}DNA}$ to produce the overexpression strains V592/UlpB-HA and V592/UlpBm-HA, complemented strains VdΔ*ulpb*/*UlpB*, VdΔ*ulpb*/*UlpBm* or Vd$^{T\text{-}DNA}$/*UlpB* respectively. pNEO-VdUlpB-GFP and pNEO-VdUlpBΔN-GFP constructs were transformed into the WT V592 to produce VdUlpB-GFP and VdUlpBΔN-GFP strains. Transformants were selected on PDA medium with 40 μg/mL G418.

To obtain the plasmids pHph-VdEno-Flag and pNAT-VdEno-GFP, the *VdEno*

gene (VDAG_03029), including the native promoter and terminator combined with the 3Flag or EGFP fragment, were amplified, and these sequences were inserted into *Hind* III/*EcoR* I-linearized pHph or pNAT binary vectors through infusion. For the pHph-VdEno$^{4K/4R}$-Flag construct, lysine residues 96, 254, 259 and 434 in the plasmid pHph-VdEno-Flag, were mutated into arginines using the Fast Site-Directed Mutagenesis kit (TIANGEN, KM101). Also, for the pHph-VdEno$^{5K/5R}$-Flag construct, lysine residue 313 in the plasmid pHph-VdEno$^{4K/4R}$-Flag, was mutated into arginine. For the pNAT-VdEno$^{5K/5R}$-GFP construct, lysine residues 96, 254, 259, 313 and 434 in the plasmid pNAT-VdEno-GFP, were mutated into arginines. These constructs were transformed into V592 to produce VdEno-Flag (named VdEno strain), VdEno-GFP, and VdEno$^{5K/5R}$-GFP strains. pNAT-VdEno-GFP construct was transformed into VdΔ*ulpb* to produce VdEno-GFP/Δ*ulpb* strain. The transformants carrying pHph vector were selected on PDA medium with 50 μg/mL hygromycin and carrying pNAT vector were selected on 50 μg/mL nourseothricin.

To obtain the overexpression plasmid pNEO-Olic-Strep-VdSUMO, the *VdSUMO* gene combined with a Strep tag were cloned and inserted into *Pac* I/*BamH* I-linearized pNEO binary vectors through infusion. The primers used above are listed in Supplemental data 1. pNEO-Strep-VdSUMO construct was transformed into V592 or VdΔ*ulpb* mutant to obtain V592/Strep-SUMO and VdΔ*ulpb*/Strep-SUMO strains. pHph-VdEno-Flag and pNEO-Strep-VdSUMO constructs were co-transformed into V592 or VdΔ*ulpb* strains to obtain VdEno/SUMO or VdEno/SUMO/Δ*ulpb* strains. pHph-VdEno$^{4K/4R}$-Flag or pHph-VdEno$^{5K/5R}$-Flag constructs were co-expressed with pNEO-Strep-VdSUMO in VdΔ*ulpb* strain to obtain VdEno$^{4K/4R}$/SUMO/Δ*ulpb* or VdEno$^{5K/5R}$/SUMO/Δ*ulpb* strains. The transformants were selected on PDA medium with 50 μg/mL hygromycin and 40 μg/mL G418.

To obtain complementary plasmid pNEO-VdSCP8-HA, the *VdSCP8* (VDAG-_08085) gene, including the native promoter and terminator together with the HA fragment were ligated into a *Hind* III/*EcoR* I-linearized pNEO binary vector. The primers used above are listed in Supplemental data 1. pNEO-VdSCP8-HA construct was transformed into the knockout mutant VdΔ*scp8* to produce the complemented strains VdΔ*scp8*/*SCP8*. The transformants were selected on PDA medium with 40 μg/mL G418. All the strains used in this study are listed in Supplemental Table 3.

Protein purification

To generate the plasmid pET-VdUlpB^{CD}, the catalytic domain of VdUlpB

(VdUlpB^{CD}, 387-780 aa) was cloned and ligated into a *BamH* I/*Xho* I-linearized pET28α vector. For the plasmid pET-VdUlpB^{CDm}, the cysteine (C711) in the catalytic domain of VdUlpB was mutated into serine using the Fast Site-Directed Mutagenesis kit (TIANGEN, KM101). All the primers used above are listed in Supplemental data 1. To purify the proteins, these plasmids were transformed into *E. coli* Rosetta (DE3), and the cultures were induced with 0.8 mM isopropyl-β-D-thiogalactopyranoside (IPTG) overnight at 16°C. The proteins were purified with Ni Sepherose™ 6 Fast Flow (GE, 17-5318-01).

To obtain the plasmids pET-Strep-pre-VdSUMO, pET-Strep-VdSUMO, pET-Strep-VdEno and pET-Strep-VdEno^{BD}, the first round PCR sequences were cloned with the primer pairs pET-SUMOHA-F1/R, pET-SUMO-F1/R, pET-Eno-F1/R and pET-Eno^{BD}-F1/R (Supplemental data 1), and the second round PCR sequences were amplified from the products with the following primer pairs pET-F2/pET-SUMOHA-R, pET-F2/pET-SUMO-R, pET-F2/pET-Eno-R and pET-F2/pET-Eno^{BD}-R (Supplemental data 1). The PCR fragments in the second round were ligated into the *BamH* I/*Xho* I-linearized pET28α vector. For the plasmid pET-Strep-VdEno^{K96R}, pET-Strep-VdEno^{K254R}, pET-Strep-VdEno^{K259R}, pET-Strep-VdEno^{K313R}, pET-Strep-VdEno^{K434R}, pET-Strep-VdEno$^{4K/4R}$ and pET-Strep-VdEno$^{5K/5R}$, the five lysines (K96, K254, K259, K313 and K434) of VdEno in pET-Strep-VdEno were substituted with arginines using the Fast Site-Directed Mutagenesis kit (TIANGEN, KM101) with the primer pairs listed in Supplemental data 1. Protein expression was performed as described above, and purification followed the method of the Strep-Tactin®XT Superflow® columns (IBA Life Sciences, 2-4013-001).

Two-dimensional electrophoresis

For total protein preparation, V592 and Vd$^{T\text{-}DNA}$ were cultured in Czapek-Dox medium, collected and lysed in extraction buffer (100 mM Tris-HCl pH 8.0, 150mM NaCl, 0.5 mM EDTA, 1% Triton X-100, 1×protease inhibitor (Roche, 04693132001)). The supernatant was extracted with Tris-saturated phenol, precipitated by methanol with 0.1 M ammonium acetate and dissolved in rehydration solution (7 M urea, 2 M thiourea, 4% CHAPS, 2% IPG buffer pH4-7 (Cytiva, Cat#17600086), 40mM DTT) for two-dimensional electrophoresis. Total proteins were quantified using 2-D Quant kit (Cytiva, Cat#80648356).

For 2-D protein separation, 800 μg of total proteins was loaded onto IPG strips (18

cm, pH 4-7; Cytiva, Cat#17123301). Isoelectric focusing (IEF) was carried out and the strips were then put on the top of SDS-PAGE gel. After electrophoresis, the protein spots were detected and quantified using Image Master 2D Platinum software (Cytiva, V6.0) and the volume ratios of corresponding spots between V592 and Vd^{T-DNA} were calculated from three biological replicates. Protein spots with a ratio higher than 2, p value <0.05 (unpaired Student's t-test) were considered significant and manually excised for mass spectrometry analysis.

Mass spectrometry analysis

To identify whether VdEno was SUMOylated, Matrix-Assisted Laser Desorption/Ionization Time-of-Flight (MALDI-TOF) mass spectrometry was performed. The excised gels and protein spots from 2D gel were digested by trypsin at 37°C overnight, and dried using a vacuum centrifuge. The dried peptides were dissolved in alpha-cyano-4-hydroxy-cinnamic acid (CHCA) MALDI matrix solution and analyzed by a MALDI-TOF/TOF mass spectrometer (4700 Proteomics Analyzer, Applied Biosystems). The combined mass spectrometry (MS) and tandem MS (MS/MS) peak lists were analyzed using Protein Pilot software (Applied Biosystems) with a Mascot search engine (MASCOT v. 2.2) and searched against the *V. dahliae* VdLs.17 protein sequence database (ASM15067v2) to identify Verticillium proteins with the following parameters: Enzyme, Trypsin; Fixed modifications, Carbamidomethyl (C); Variable modifications, Oxidation (M); Mass values: MONOISOTOPIC; Peptide Mass Tolerance: ± 100 ppm; Fragment Mass Tolerance: ± 0.6 Da; Max Missed Cleavages: 1. Ions score is -10*Log(P), where P is the probability that the observed match is a random event. Individual ions score > 26 indicate identity or extensive homology ($p < 0.05$). Protein scores are derived from ions scores as a non-probabilistic basis for ranking protein hits.

EMSA

The purified conserved DNA binding domain of VdEno (VdEno^{BD}) was incubated with specific probes for 1 h at room temperature. The competition experiment was conducted by adding a 50-fold molar excess of cold probes or nonspecific probes into the reaction before adding the specific probes. The products were analyzed with a 4% native PAGE gel and transferred to a HybondTM-N$^+$ membrane (GE, RPN303B). The probes were amplified with the primer pairs listed in Supplemental data 1, and they were labeled with ^{32}P as described above.

Chromatin immunoprecipitation (ChIP)

Conidia and mycelia of *V. dahliae* were cultured in liquid Czapek-Dox medium for 3 days and treated with 1% formaldehyde for 10 min at room temperature, followed by the addition of glycine to a final concentration of 0.125 M, and this was done for 5 min to arrest the cross-linking reaction. The cultures were collected and lysed with ChIP buffer (10 mM Tris pH 8.0, 150 mM NaCl, 0.5 mM EDTA, 1% Triton X-100, 1×Protease inhibitor). Chromatin was sheared by sonication on ice and centrifuged at 17949 g for 15 min at 4°C. Ten percent of the supernatant was used as input, while the remaining supernatant was incubated with anti-IgG or anti-GFP agarose beads overnight at 4°C. After washing, the immunoprecipitated material was eluted from beads by SDS sample buffer (1% SDS, 100 mM $NaHCO_3$) for 1 h at 65°C. The elution was treated with RNase A and Proteinase K for 6 h at 65°C, and the DNA that had bound with VdEno was extracted with the Gene JET PCR Purification Kit (Thermo Scientific, K0702). qPCR assays were performed to detect the binding ability of VdEno to the *VdSCP8* promoter. The primers used for ChIP-qPCR are listed in Supplemental data 1.

Quantitative real-time PCR

To examine the transcript pattern of *VdUlpB*, *VdUbc9*, *VdSCP8* and *VdEno*, cotton roots infected with *V. dahliae* V592 at different times were collected. To examine the transcript level of *VdSCP1-10* and *VdSCP41*, conidia from V592 and Vd*Δulpb* strains were collected. Total RNA was extracted with a Plant Total RNA Purification Kit (Gene Mark, TR02-150). cDNA was synthesized with HiScript II QRT SuperMix (Vazyme, R223-01), and qPCR was conducted with ChamQ Universal SYBR qPCR Master Mix (Vazyme, Q711-02) using a CFX96 real-time system (Bio-Rad). The primers are listed in Supplemental data 1.

Promoter-luciferase Assay

Promoter activity assays were performed in *Nicotiana benthamiana* leaves. A construct pCAMBIA 1300-221-35S-6×Myc expressing VdEno fusion protein was co-injected with the constructs pLOCex which luciferase was driven by 35S, *VdSCP1*, *VdSCP7*, *VdSCP8*, *VdSCP9*, *VdSCP10*, *VdSCP41* or *VdSCP8* mut promoters. Empty vector was co-injected with the constructs pLOCex used as negative control. After 2d agroinfiltration, leaves were collected and D-luciferin was added to observe under Tanon 4600 and Image J software (V2.0.0) was used to measure the expression level of luciferase. The primers are listed in Supplemental data 1. VdEno-Myc was detected

by Western blotting with anti-c-Myc antibody (EASYBIO, BE3203-100) with a 1:5000 dilution.

Supplemental Figures

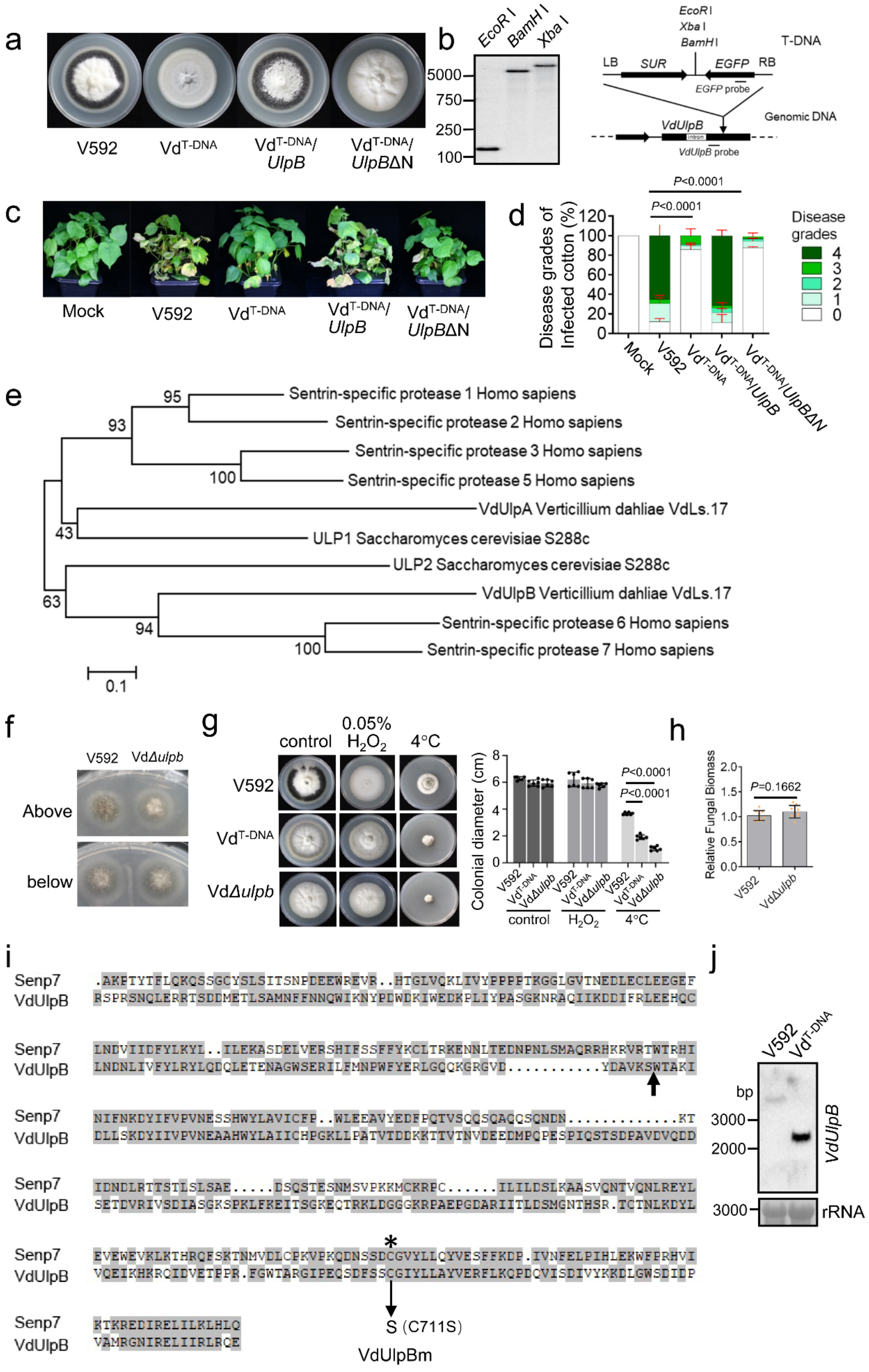

Supplementary Fig. 1. Identification of the Vd$^{T\text{-}DNA}$ mutant.

(a) Colony morphology of wild-type V592, T-DNA insertion mutant Vd$^{T\text{-}DNA}$ and complementary strains: Vd$^{T\text{-}DNA}$/*UlpB* and Vd$^{T\text{-}DNA}$/*UlpB*ΔN, on PDA plates after 19 dpi.

(b) Identification of Vd$^{T\text{-}DNA}$ by Southern blotting (*Left*) and TAIL-PCR (*Right*). Genomic DNA was digested with three restriction enzymes and incubated with an *EGFP* probe.

(c) Disease symptoms of cotton infected with V592, Vd$^{T\text{-}DNA}$, Vd$^{T\text{-}DNA}$/*UlpB* or Vd$^{T\text{-}DNA}$/*UlpB*ΔN at 22 dpi.

(d) Disease grades were evaluated with three replicates of 36 plants for each inoculum (mean ± s.d., t-test, two-sided).

(e) Phylogenetic analysis of SUMO-specific proteases in humans, yeast and *V. dahliae*.

(f) Penetration assay. The VdΔ*ulpb* mutant penetrated the cellophane membrane similar to V592. Photographs show fungus grown on a cellophane membrane laid on MM (above, at 7 dpi) and after removal of the cellophane membrane (below).

(g) H_2O_2 and cold stress response assay with V592, Vd$^{T\text{-}DNA}$ and VdΔ*ulpb* strains. Colony morphology of the indicated strains on potato dextrose agar (PDA) plates (*Left*). Statistical analysis of growth under H_2O_2 or cold stress condition (*Right*). Strains without stress or under H_2O_2 stress were cultured for 20 days, at 4℃ for 55 days (mean ± s.d., n = 6 biologically independent samples, one-way ANOVA followed by Tukey's multiple comparisons test).

(h) Fungal biomass in VdΔ*ulpb*-infected cotton plants similar to V592-infected ones at 5 dpi. The values were qPCRof fungal internal transcribed spacer DNA relative to cotton GhUBQ7 (GeneBank: DQ116441) DNA. (mean ± s.d.; n=9 biologically independent samples, t-test, two-sided).

(i) Analysis of the T-DNA insertion site by aligning the sequences of SENP7 and VdUlpB (full-length: 1063aa). The arrow indicates the location of T-DNA insertion.

The asterisk indicates the cysteine at residue 711 (C711) in the protease catalytic domain (within 387-780 aa of VdUlpB). VdUlpBm is a C-to-S mutation (C711S).

(j) Expression pattern of *VdUlpB* in V592 and Vd$^{T\text{-}DNA}$ by Northern blotting. The probe amplified from the coding sequence of *VdUlpB* which was indicated in b.

The experiments in **b, f, j** were repeated independently three times with similar results. Source data are provided as a Source Data file.

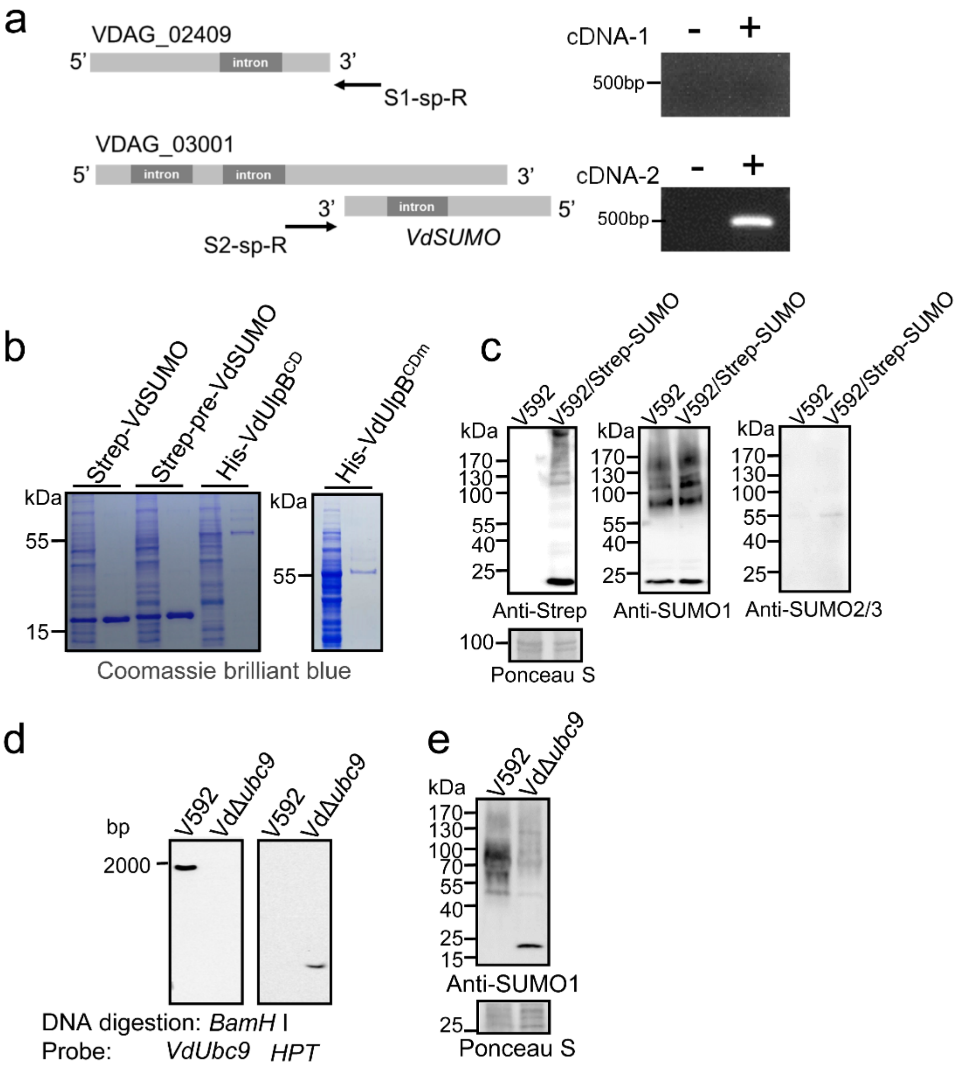

Supplementary Fig. 2. Identification of *VdSUMO*, purification of proteins and detection of SUMOylation in *V. dahliae*.

(a) Detection of transcription of *VdSUMO* by reverse transcription PCR. Arrows indicated the location of specific reversed primers (S1-sp-R and S2-sp-R) designed for

two putative *VdSUMO* loci. Transcript was obtained from VDAG_03001 and named as *VdSUMO*.

(b) Coomassie brilliant blue staining of purified proteins. Proteins fused with His-Tag or Strep-Tag were purified using Ni Sepherose™ 6 Fast Flow or Strep-Tactin®XT Superflow® columns, respectively.

(c) Detection of VdSUMO and SUMOylated proteins *in vivo*. The Strep-VdSUMO construct was transformed into WT V592, and total protein was extracted followed by Western blot with anti-Strep, anti-human SUMO1 or anti-human SUMO2/3 antibody. Ponceau staining was used as a loading control.

(d) Conformation of the knockout mutant VdΔ*ubc9* by Southern blotting. The probes were labeled with biotin.

(e) Detection of the whole protein SUMOylation level in the SUMO ligase VdUbc9 mutant (VdΔ*ubc9*) and WT V592. Ponceau staining was used as a loading control.

The experiments in **a**, **b**, **c**, **d**, **e** were repeated independently three times with similar results. Source data are provided as a Source Data file.

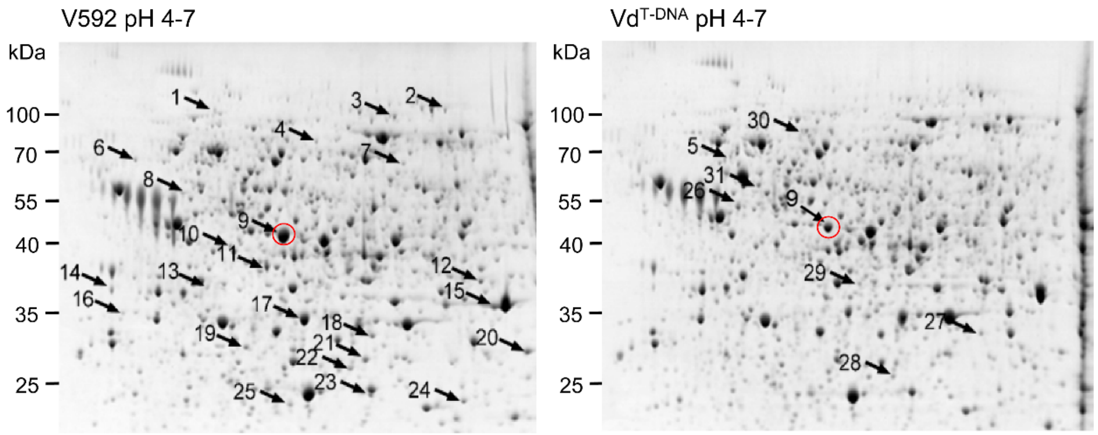

Supplementary Fig. 3. Determination of potential VdUlpB substrates in *V. dahliae*. Proteome analysis of V592 and Vd^{T-DNA}. Total proteins from V592 and Vd^{T-DNA} were extracted and separated by two-dimensional gel electrophoresis. The proteins (pointed by arrows 1-31) differentially expressed between the two strains were identified by MS. Red circle indicates VdEno which was evidently decreased in Vd^{T-DNA}. The

experiments were repeated independently three times with similar results. Source data are provided as a Source Data file.

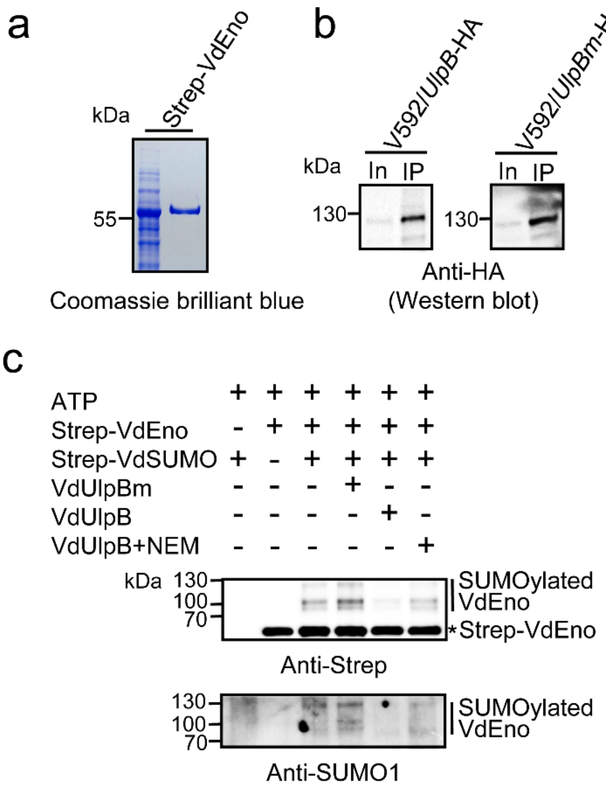

Supplementary Fig. 4. Identification of the catalytic activity of full-length VdUlpB.

(a) Coomassie brilliant blue staining of purified VdEno. VdEno fused with Strep-Tag were purified using Strep-Tactin®XT Superflow® columns.

(b) Identification the expression of VdUlpB in V592/*UlpB*-HA and V592/*UlpBm*-HA strains by Western blotting with anti-HA antibody. Total protein was extracted (In) and immunoprecipitated with anti-HA agarose beads (IP).

(c) Identification of the catalytic activity of full-length VdUlpB. VdEno was SUMOylated by VdSUMO, and incubated with the immunoprecipitated VdUlpBm, VdUlpB or NEM treated VdUlpB at room temperature for 4 h followed by Western blotting with anti-Strep or anti-human SUMO1 antibody.

The experiments in **a**, **b**, **c** were repeated independently three times with similar results. Source data are provided as a Source Data file.

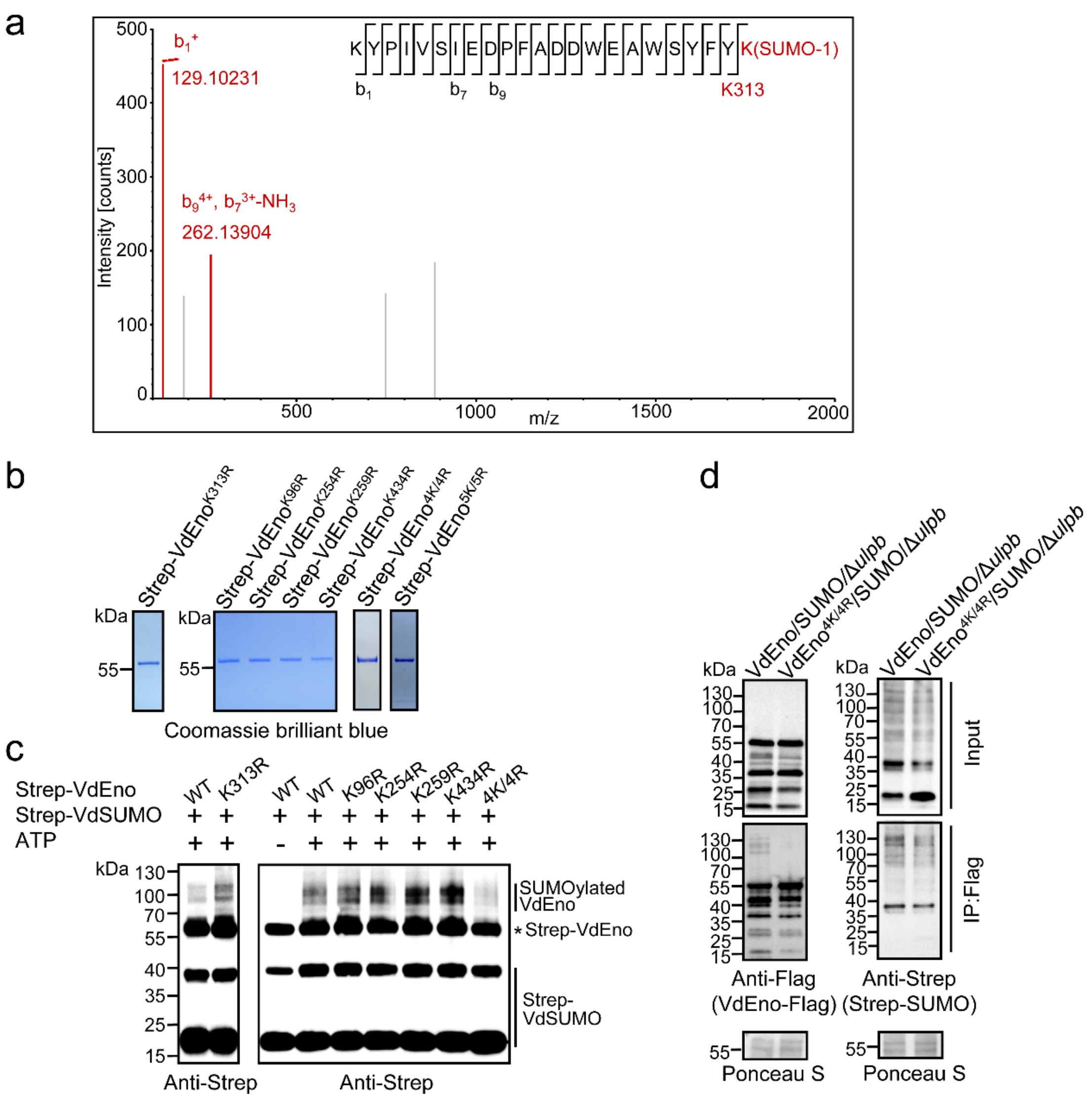

Supplementary Fig. 5. Identification of VdEno SUMOylation sites.

(a) VdEno SUMOylation sites were identified by LC-MS/MS. SUMOylated VdEno was immunoprecipitated from VdEno/SUMO/Δ*ulpb* strain and separated by SDS PAGE gel, and SUMOylation sites was analyzed by AIMS company.

(b) Coomassie brilliant blue staining of purified proteins. Proteins fused with Strep-Tag were purified using Strep-Tactin®XT Superflow® columns.

(c) The lysine of 313 site (identified by LC-MS/MS shown in a) was mutated into arginine, and the lysines of 96, 254, 259 and 434 sites in VdEno were individually or simultaneously mutated into arginines. The SUMOylation level was compared to WT VdEno *in vitro*.

(d) Conformation of VdEno SUMOylation residues *in vivo*. VdEno-Flag or VdEno$^{4K/4R}$ -Flag constructs were co-expressed with Strep-VdSUMO in the VdΔ*ulpb* mutant. Total protein from these samples was immunoprecipitated with anti-Flag beads and immunoblotted with anti-Flag or anti-Strep antibody. Ponceau staining was used as a loading control.

The experiments in **b**, **c**, **d** were repeated independently three times with similar results. Source data are provided as a Source Data file.

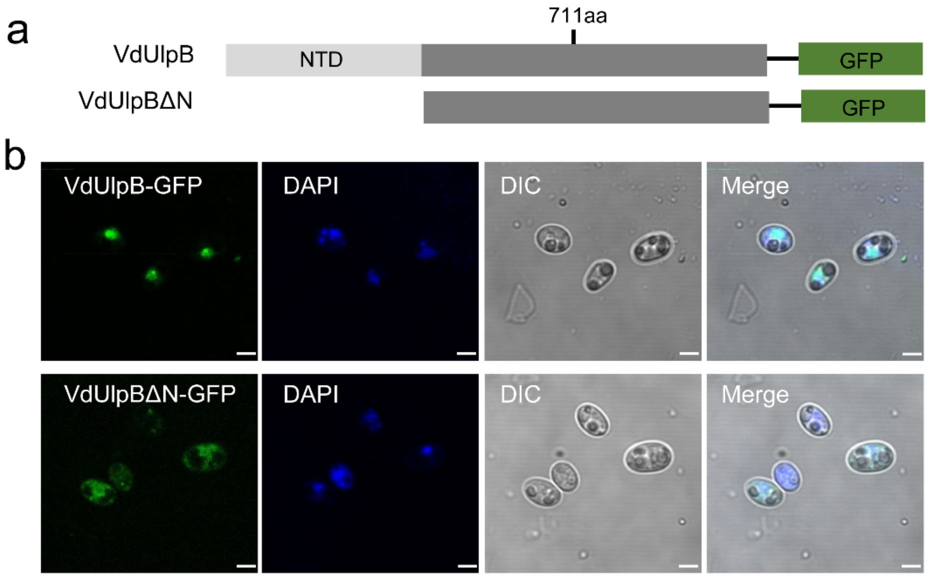

Supplementary Fig. 6. The N-terminal domain of VdUlpB is essential for its nuclear localization.

(a) The schematic diagram of full-length and N-terminal deletion of VdUlpB (VdUlpBΔN) with a GFP epitope tag. C711 indicated the catalytic site of VdUlpB (upper panel).

(b) Localization of the full-length WT VdUlpB and VdUlpBΔN were observed under confocal laser scanning microscopy (CLSM). Scale bar = 3 μm (lower panel). The experiments were repeated independently three times with similar results.

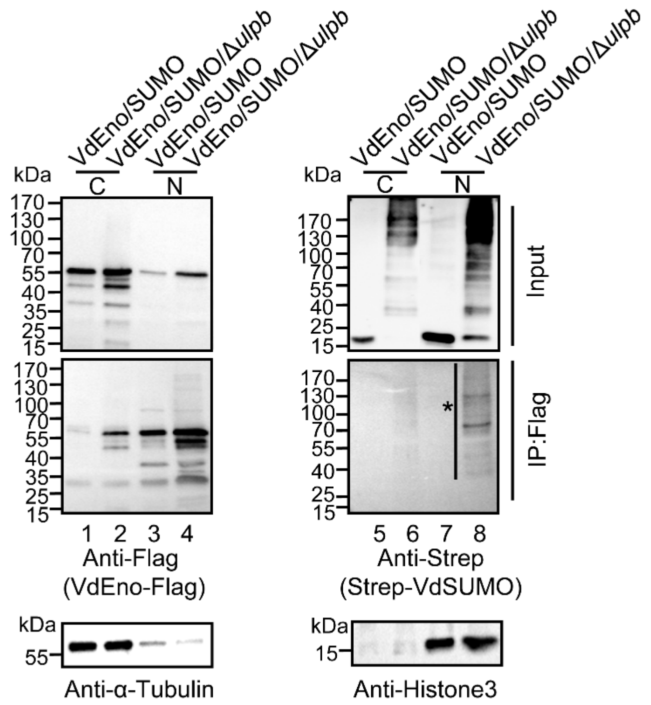

Supplementary Fig. 7. SUMOylation affects VdEno localization.

Separation of the nuclear and cytoplasmic VdEno from the VdEno/SUMO and VdEno/SUMO/Δ*ulpb* strains. Nuclear (N) and cytoplasmic (C) sections were separated. Anti-histone3 and anti-α-tubulin antibody, the nuclear and cytoplasmic markers, respectively, were used as indications. Immunoprecipitation with anti-Flag beads from the nuclear and cytoplasmic sections followed by Western blotting with anti-Flag or anti-Strep antibody. Asterisk indicates the SUMOylated VdEno. The experiments were repeated independently three times with similar results. Source data are provided as a Source Data file.

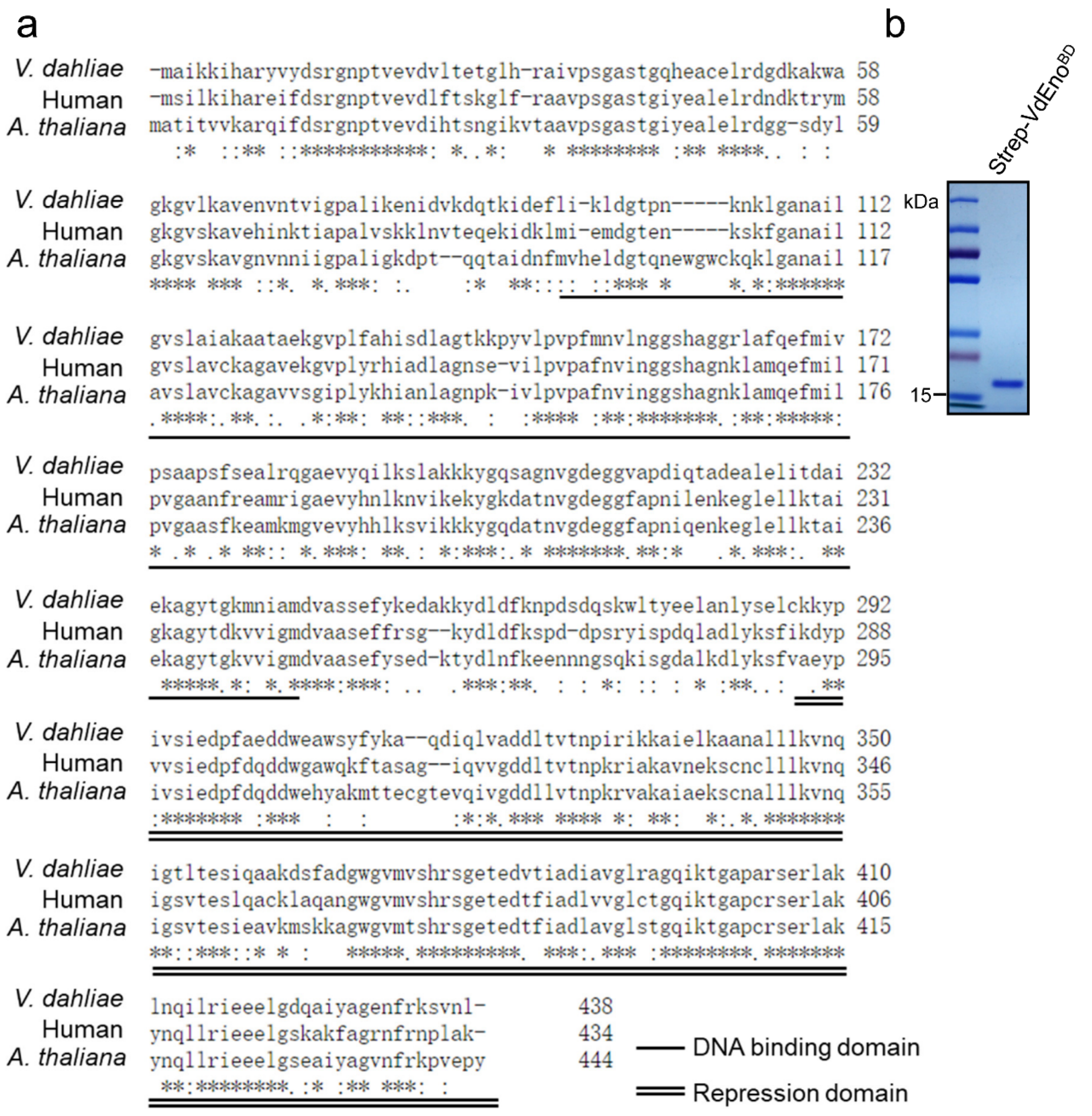

Supplementary Fig. 8. Amino acid sequence alignments of enolases from *V. dahliae*, human and *A. thaliana*.

(a) The DNA-binding domain is underlined and the transcriptional repression domain is double-underlined.

(b) Coomassie brilliant blue staining of purified the DNA-binding domain of VdEno ($VdEno^{BD}$) protein. $VdEno^{BD}$ fused with Strep tag was purified using Strep-Tactin®XT Superflow® columns. The experiments were repeated independently three tsimilar results. Source data are provided as a Source Data file.

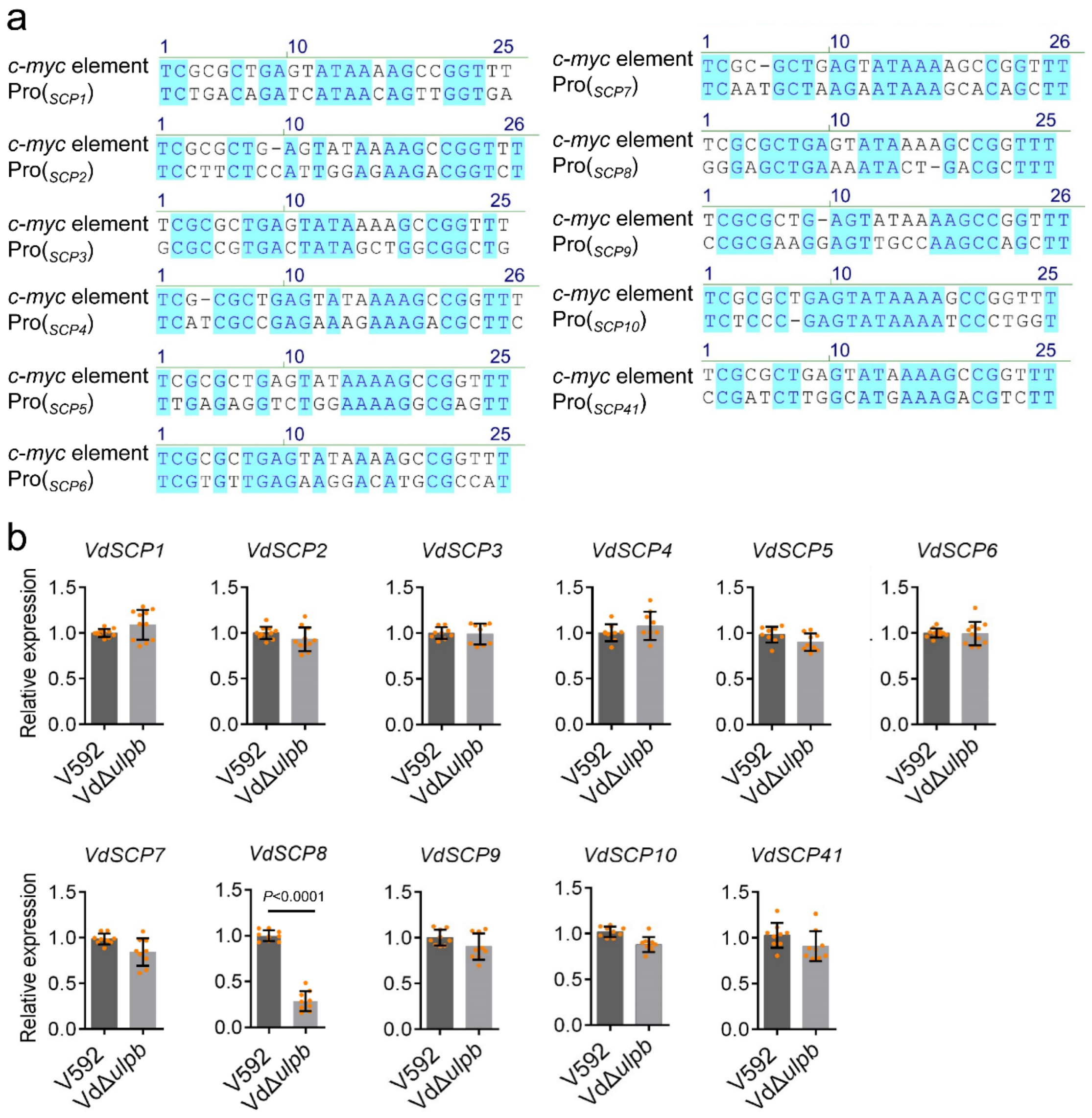

c 5'-CTGCTCGTTCGGGCCTGCTCGTGTGCTTATCAGCCAGGAACCAGGTACCA
TGGGAGCTGAAAATACTGACGCTTTCGTTTGTTGAAGGCGGCGCTGTTGT-3'

Supplementary Fig. 9. Alignments of human enolase binding element with promoters of tested *V. dahliae* genes and detection of their expression.

(a) Putative hit promoter regions with "*c-myc* promoter element". Sequence alignments of human enolase-bound "*c-myc* promoter element" and promoters of genes known encoding secretory proteins (VdSCPs).

(b) Relative expression of *VdSCP* genes in WT V592 and VdΔ*ulpb* strains (mean ± s.d., $n = 9$ individual data points are shown, t-test, two-sided). The values of each transcript from V592 relative to internal control *elf1*-α was arbitrarily designated as 1. Results

were obtained from three biologically independent samples. Standard deviations are indicated by the error bars.

(c) The 100 bp end-labeled sequence from *VdSCP8* promoter containing the "*c-myc* promoter element" hit region was used for EMSA with VdEno^{BD} (in Figure 6a). Source data are provided as a Source Data file.

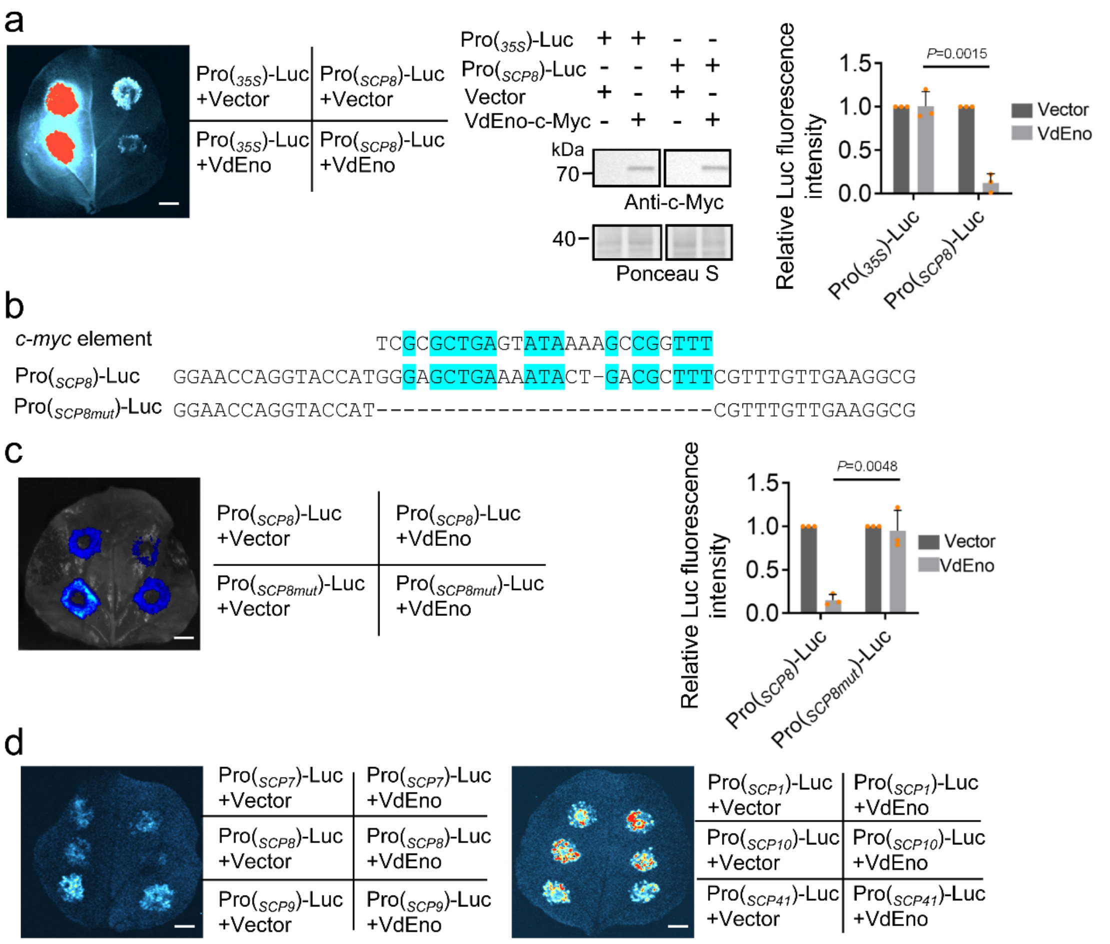

Supplementary Fig. 10. Detection of VdEno-repressed *VdSCP8* expression in a plant transient expression system.

(a) VdEno-dependent repression on *VdSCP8* promoter in *Nicotiana benthamiana* leaves. Detection of Luciferase (Luc) activity by agroinfiltration-mediated co-expression of *35S-VdEno-Myc* or empty vector with *Luc* gene driven under *35S* promoter or *VdSCP8* promoter. Scale bar=1 cm. VdEno-Myc was detected by Western blotting with anti-c-Myc antibody. Statistical analysis of Luc activities by Image J software, the values with empty vector were arbitrarily designated as 1. At least three

experiments were performed (mean ± s.d., n = 3 biologically independent samples, t-test, two-sided).

(b) Sketch of alignment sequences of *VdSCP8* promoter (Pro(*SCP8*)-Luc) or *VdSCP8* mutant promoter lacking the human "*c-myc* promoter element" hit region (Pro(*SCP8mut*)-Luc) that were used to drive *Luc* gene.

(c) VdEno-dependent repression on Pro(*SCP8*) but not on Pro(*SCP8mut*). Detection of VdEno-repressed Luc activity was as described in (a) (mean ± s.d., n = 3 biologically independent samples, t-test, two-sided). Scale bar=1 cm.

(d) Detection of effect of VdEno on other *VdSCP* gene promoters. Luc gene was driven under *VdSCP7*, *VdSCP8*, *VdSCP9*, *VdSCP1, VdSCP10* or *VdSCP41* promoters and co-expression with *35S-VdEno* or empty vector as indicated. Except for Pro(*SCP8*)-Luc, VdEno-dependent repression on Luc activity was not observed compared to co-expression with vector. Scale bar=1 cm. The experiments were repeated independently three times with similar results.

Source data are provided as a Source Data file.

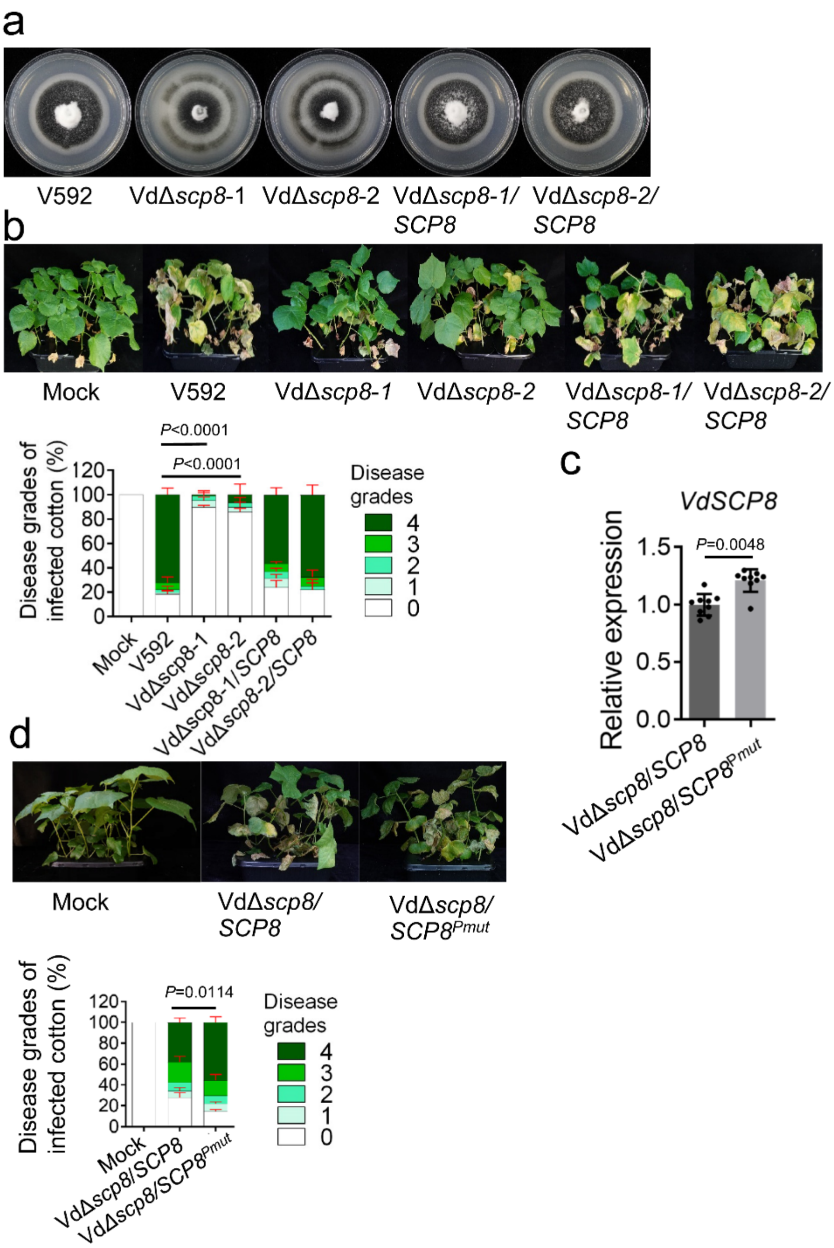

Supplementary Fig. 11. VdSCP8 functions as an effector.

(a) Colony morphology of the WT V592, mutant VdΔ*scp8-1/2* and complementary strain VdΔ*scp8/SCP8-1/2* on PDA plates for 17 dpi.

(b, d) Disease symptoms and disease grades of cotton infected with the indicated strains at 22 dpi as described in Figure 1. The disease grades were evaluated with three replicates of 36 plants for each inoculum (mean ± s.d., t-test, two-sided).

(c) Relative expression of *VdSCP8* in the VdΔ*scp8/SCP8* and VdΔ*scp8/SCP8^{Pmut}* strains (mean ± s.d., $n = 9$ individual data points are shown, t-test, two-sided). The

values of each transcript from VdΔ*scp8/SCP8* relative to the internal control *elf1*-α were arbitrarily designated as 1. Source data are provided as a Source Data file.

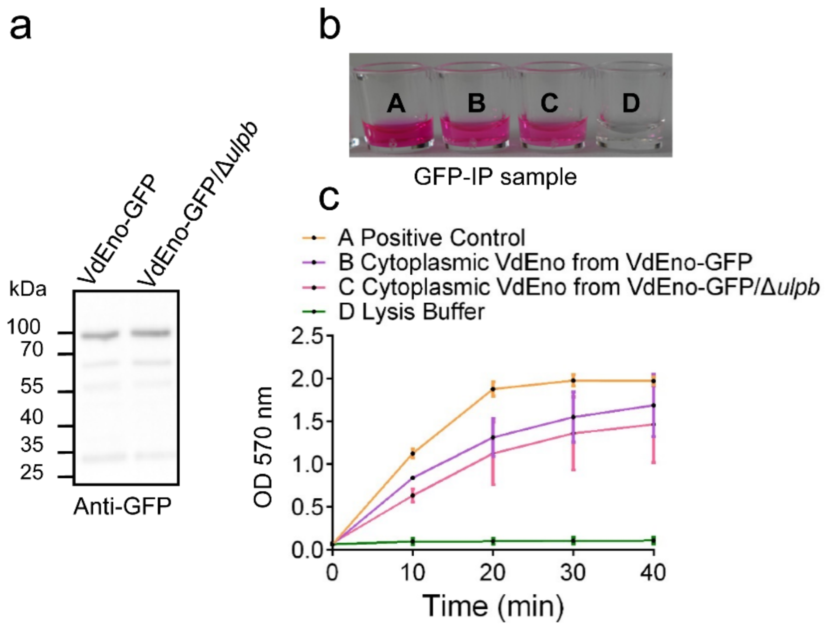

Supplementary Fig. 12. Detection of glycolytic activity of cytoplasmic VdEno in VdEno-GFP and VdEno-GFP/Δ*ulpb* strains.

(a) Protein detection by Western blotting with Anti-GFP. Equal amounts of cytoplasmic IPed-VdEno proteins from the VdEno-GFP and VdEno-GFP/Δ*ulpb* strains were used.

(b) Detection of glycolytic activity of the cytoplasmic IPed-VdEno proteins from the VdEno-GFP (sample B) and VdEno-GFP/Δ*ulpb* (sample C) strains. Sample A was a positive control and sample D was a buffer control. The glycolytic activity was detected by the method described in Fig 5b. The experiments were repeated independently three times with similar results.

(c) Analysis of the glycolytic activity of IPed-VdEno (mean ± s.d., n = 2 biologically independent samples).

Source data are provided as a Source Data file.

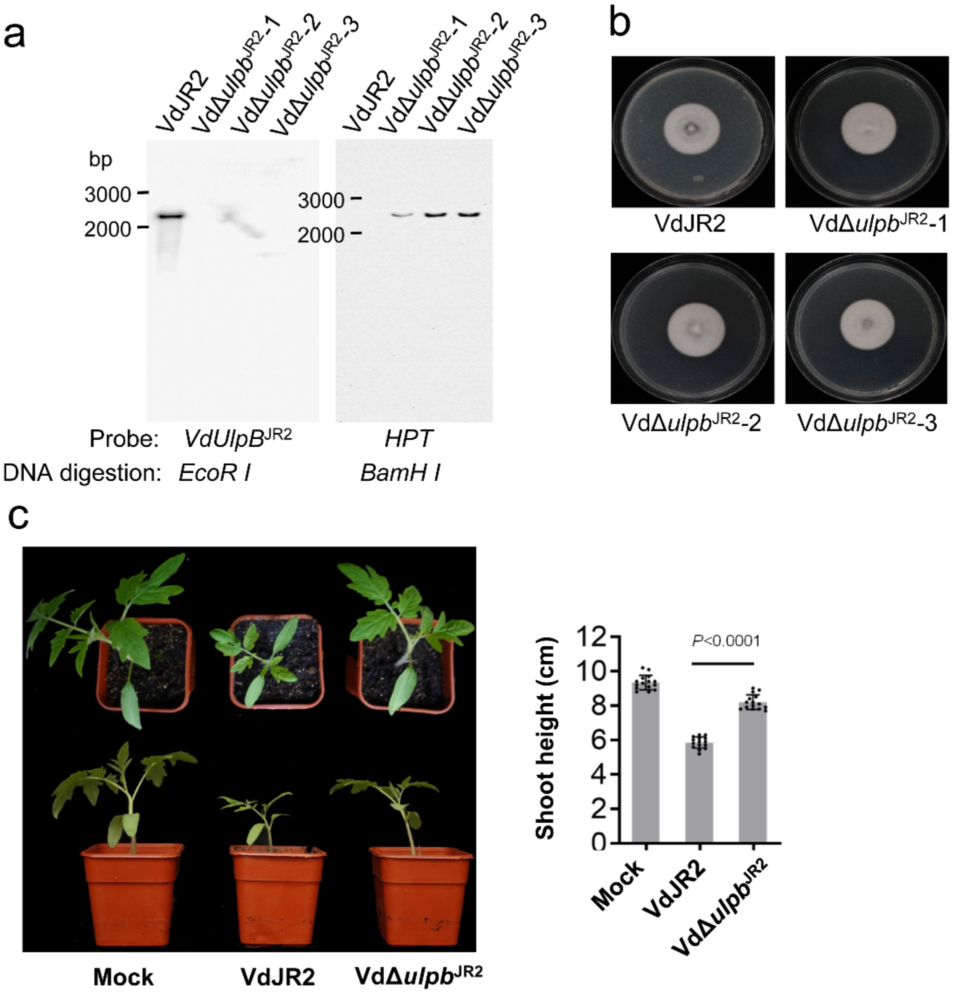

Supplementary Fig. 13. VdUlpB is required for pathogenicity of VdJR2 on tomato plants.

(a) Confirmation of the knockout mutant VdΔ*ulpb*^{JR2} by Southern blotting with the indicated probes. Genomic DNA was digested by EcoR I and BamH I. The experiments were repeated independently three times with similar results.

(b) Colony morphology of WT VdJR2 and VdΔ*ulpb*^{JR2} on PDA plates at 10 days. The experiments were repeated independently three times with similar results.

(c) Disease symptoms of tomato plants infected with the indicated strains at 12 dpi (mean ± s.d., n = 15 individual data points are shown, one-way ANOVA followed by Tukey's multiple comparisons test). Source data are provided as a Source Data file.

Supplemental Tables

Supplementary Table 1. IPed-MS results with anti-Strep in VdΔ*ulpb*/Strep-SUMO strain.

NO.	Accession No.	Description	Coverage (%)
**1**	EGY14431.1	Uncharacterized protein	23.58
**2**	EGY20885.1	Ubiquitin-like domain-containing protein	19.39
**3**	EGY23328.1	60S ribosomal protein L13	18.69
**4**	EGY18111.1	Actin (Fragment)	16.05
**5**	EGY21589.1	Enolase_C domain-containing protein	12.63
**6**	EGY19520.1	H15 domain-containing protein	10.15
**7**	EGY15713.1	Uncharacterized protein	9.81
**8**	EGY21035.1	Translation elongation factor 1-alpha (Fragment)	9.58
**9**	EGY13590.1	Outer mitochondrial membrane protein porin	9.54
**10**	EGY20653.1	Pyruvate carboxylase	7.41
**11**	EGY19081.1	Histone H2B OS	6.57
**12**	EGY16371.1	ADP, ATP carrier protein	5.81
**13**	EGY14352.1	Uncharacterized protein	4.8
**14**	EGY23113.1	Antigen 1	4.76
**15**	EGY23139.1	RRM domain-containing protein	4.68
**16**	EGY14888.1	40S ribosomal protein S3	4.58
**17**	EGY21847.1	Uncharacterized protein	4.55
**18**	EGY22948.1	Uncharacterized protein	3.53
**19**	EGY14751.1	Uncharacterized protein	3.08
**20**	EGY22134.1	Advillin	1.57
**21**	EGY20788.1	Cell division cycle protein	1.35

Supplementary Table 2. Proteins with different amount between V592 and Vd^{T-DNA}.

Spot No.	Accession No.	Protein name	Changed amount in Vd^{T-DNA}
**1**	EGY22745.1	carbamoyl-phosphate synthase arginine-specific large chain	down
**2**	EGY20389.1	2-oxoglutarate dehydrogenase E1	down
**3**	EGY18821.1	glycogen phosphorylase	down
**4**	EGY20678.1	clathrin heavy chain	down
**5**	EGY23207.1	heat shock protein	up
**6**	EGY22689.1	calnexin	down
**7**	EGY15515.1	fimbrin	down
**8**	EGY17971.1	nucleosome assembly protein	down
**9**	EGY21589.1	enolase	down
**10**	EGY22944.1	cell division control protein	down
**11**	EGY18111.1	actin	down
**12**	EGY17633.1	formate dehydrogenase	down
**13**	EGY16347.1	succinyl-CoA ligase beta-chain	down
**14**	EGY17143.1	vacuolar protease A	down
**15**	EGY23312.1	succinyl-CoA ligase subunit alpha	down
**16**	EGY15643.1	hypothetical protein VDAG_06807	down
**17**	EGY16047.1	pyrimidine precursor biosynthesis enzyme THI11	down
**18**	EGY16767.1	retinol dehydrogenase	down
**19**	EGY18798.1	chaperone protein hchA	down
**20**	EGY21146.1	cerevisin	down
**21**	EGY15967.1	proteinase R	down
**22**	EGY21676.1	cytochrome c peroxidase	down
**23**	EGY21555.1	adenylyl-sulfate kinase	down
**24**	EGY16389.1	NAD dependent epimerase/dehydratase family protein	down
**25**	EGY18022.1	elongation factor 1-gamma 1	down
**26**	EGY16673.1	26S protease regulatory subunit 6A	up
**27**	EGY17776.1	elongation factor Tu	up
**28**	EGY23040.1	ATP synthase gamma chain	up
**29**	EGY19103.1	RNP domain-containing protein	up
**30**	EGY21633.1	Cobalamin-independent synthase	up
**31**	EGY21724.1	hypothetical protein VDAG_03164	up

Supplementary Table 3. Strains used in this study.

Strains	Genotype description	References
V592	Wild type	Gao et al., 2010 [1]
Vd^{T-DNA}	*VdUlpB* insertion mutant	Gao et al., 2010 [1]
Vd^{T-DNA}/*UlpB*	Vd^{T-DNA} complementation with *VdUlpB*	This study
Vd^{T-DNA}/*UlpBΔN*	Vd^{T-DNA} complementation with *VdUlpBΔN* deletion of *VdUlpB* in V592	This study
VdΔ*ulpb*	deletion of *VdUlpB* in V592	This study
VdΔ*ulpb*/*UlpB*	VdΔ*ulpb* complementation with *VdUlpB*	This study
VdΔ*ulpb*/*UlpBm*	VdΔ*ulpb* complementation with *VdUlpBm*	This study
V592/Strep-SUMO	Transformant of V592 expressing Strep-SUMO	This study
VdΔ*ubc9*	deletion of *VdUbc9* in V592	This study
VdΔ*ulpa*	deletion of *VdUbcA* in V592	This study
VdΔ*ulpb*/*UlpB*-HA/Eno-TurboID	Transformant of VdΔ*ulpb*/*UlpB*-HA expressing VdEno-TurboID	This study
VdΔ*ulpb*/*UlpBm*-HA/Eno-TurboID	Transformant of VdΔ*ulpb*/*UlpBm*-HA expressing VdEno-TurboID	This study
VdEno	Transformant of V592 expressing VdEno-Flag	This study
VdEno/SUMO	VdEno-Flag and Strep-VdSUMO constructs were co-expressed into V592	This study
VdEno/SUMO/Δ*ulpb*	VdEno-Flag and Strep-VdSUMO constructs were co-expressed into VdΔ*ulpb* strain	This study
VdEno$^{4K/4R}$/SUMO/Δ*ulpb*	VdEno$^{4K/4R}$-Flag and Strep-VdSUMO constructs were co-expressed into VdΔ*ulpb* strain	This study
VdEno$^{5K/5R}$/SUMO/Δ*ulpb*	VdEno$^{5K/5R}$-Flag and Strep-VdSUMO constructs were co-expressed into VdΔ*ulpb* strain	This study
VdEno-GFP	Transformant of V592 expressing VdEno-GFP	This study
VdEno$^{5K/5R}$-GFP	Transformant of V592 expressing VdEno$^{5K/5R}$-GFP	This study
VdEno-GFP/Δ*ulpb*	*VdUlpB* deletion in VdEno-GFP strain	This study
VdEno-GFP/Δ*ulpb*/*UlpB*	VdEno-GFP/Δ*ulpb* complementation with *VdUlpB*	This study
VdΔ*scp8*	deletion of *VdSCP8* in V592	Zhang et al., 2017 [2]
VdΔ*scp8*/*SCP8*	VdΔ*scp8* complementation with *VdSCP8*	This study
VdΔ*ulpb*/Strep-SUMO	Transformant of VdΔ*ulpb* expressing Strep-VdSUMO	This study
VdJR2	Wild type	This study
VdΔ*ulpb*^{JR2}	deletion of *VdUlpB* in JR2	This study

Supplementary References

1. Gao F*, et al.* A glutamic acid-rich protein identified in *Verticillium dahliae* from an insertional mutagenesis affects microsclerotial formation and pathogenicity. *PloS one* **5**, e15319 (2010).
2. Zhang L*, et al.* The *Verticillium*-specific protein VdSCP7 localizes to the plant nucleus and modulates immunity to fungal infections. *The New phytologist* **215**, 368-381 (2017).
